# Dependence of arterial input function on position in the left ventricle and time in the cardiac cycle

**DOI:** 10.1186/1532-429X-11-S1-P213

**Published:** 2009-01-28

**Authors:** Nicholas S DuRocher, Elodie Breton, Sohae Chung, Daniel Kim, Leon Axel

**Affiliations:** NYU Langone Medical Center – Center for Biomedical Imaging, New York, NY USA

**Keywords:** Aortic Valve, Cardiac Cycle, Aortic Root, Arterial Input Function, Aortic Valve Close

## Introduction

Determination of the arterial input function (AIF) is important in assessing myocardial perfusion with first pass perfusion cardiac MRI (CMR). While the signal-time curve measured at the aortic root level is likely to be the best estimate of the AIF to the left ventricle (LV) wall, because of proximity to the coronary artery openings, most studies determine the AIF from the blood pool inside the LV, since it does not require any additional data acquisition. Previous studies did not report significant differences in AIFs measured at different LV/aorta positions or at different times of the cardiac cycle [1, 2]. However, we have observed differences when analyzing the AIF in certain studies and sought to investigate further. In this work we investigated if there were significant differences in the measured AIF depending on where in the LV it was measured, and on when during the cardiac cycle it was measured.

## Methods

First-pass perfusion MRI, using saturation-recovery TurboFLASH readouts with the following parameters: FOV = 340 × 255 mm; Matrix = 64 × 48; TE/TR = 1.12/2.3 ms; TD = 12.4 ms; Image Acquisition Time = 55.2 ms; Total Acquisition Time = 76.2 ms, was performed 3 times in a healthy volunteer in a 3 T whole-body MR scanner (Tim Trio, Siemens, Germany). Immediately following a 5 mL bolus injection of Gd-DTPA (0.5 M) into the right antecubital vein, 8 to 10 identically positioned long axis three-chamber view images were successively acquired in every cardiac cycle. In the resulting images, regions of interest (ROIs) were manually drawn at 6 different locations in the heart: left atrium, base level close to the mitral valve, base level close to the aortic valve, total base level, apex of the left ventricle, and aortic root. The time-to-peak (TTP) of the signal intensity curve was independently assessed at every time after the QRS (cardiac phase) and in every location.

## Results

**Figure 1:**
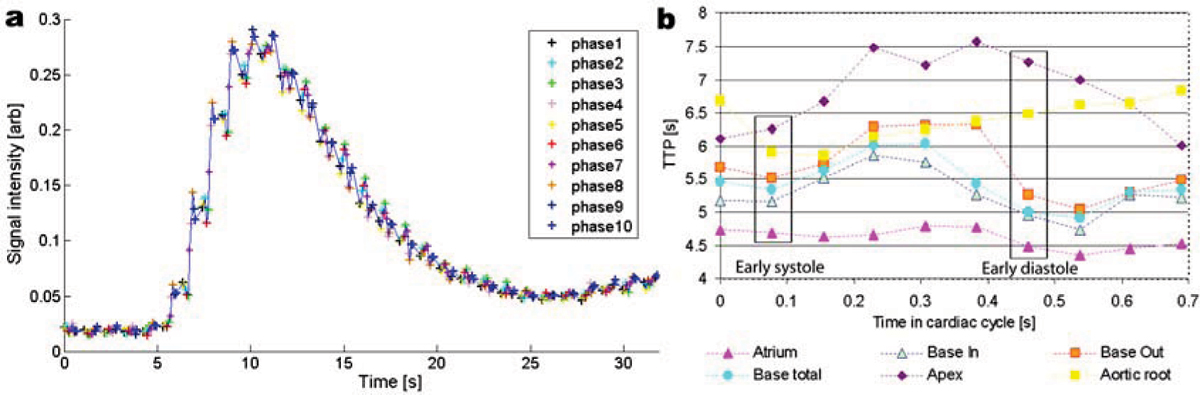
Figure 1

Figure 1a shows a typical signal-time curve obtained at the base level when plotting all the cardiac phase acquisitions together. This curve shows an oscillation during the cardiac cycle superimposed on the main contrast enhancement curve. The TTP obtained for the 6 different LV areas are represented in Figure 1b, separately for phase when the data have been acquired. Following the QRS complex, the heart contracts and the blood is ejected, making TTP's in the aortic root and at the base level close to the aortic valve similar in systole. During diastole, the aortic valve closes, and while the contrast levels remain relatively constant in the aortic root, the TTP inside the LV changes, reflecting ventricular filling. The TTP at the apex is approximately 1 s longer than the base TTP in systole, and it increases to about 2 s longer in early diastole.

## Conclusion

In a healthy volunteer, we found that at every location considered (atrium/LV/aorta), the contrast enhancement curve fluctuates, depending on when the data have been acquired in the cardiac cycle. Moreover, the AIFs measured at the base and apex levels have significantly different time courses, as reflected in their TTP, which might result in different calculations of cardiac perfusion, depending on which is used as the AIF. We found that the signal-time curve at the base level near the aortic valve is a good approximation of the aortic root AIF in systole, but not in diastole. Further experiments are required in order to study the influence of these time differences in AIF curves, relative to the acquisition's localization and time in the cardiac cycle, on the analysis of first pass perfusion CMR.
